# Factors associated with myopia in Korean children: Korea National Health and nutrition examination survey 2016–2017 (KNHANES VII)

**DOI:** 10.1186/s12886-020-1316-6

**Published:** 2020-01-20

**Authors:** Hyuna Kim, Jin Seok Seo, Woong-Sun Yoo, Gyu-Nam Kim, Rock Bum Kim, Jae Eun Chae, Inyoung Chung, Seong-Wook Seo, Seong Jae Kim

**Affiliations:** 1 Department of Ophthalmology, Gyeongsang National University School of medicine and Gyeongsang National University Hospital, Jinju, South Korea; 2 Gyeongsang Institute of Health Science, Gyeongsang National University, Jinju, South Korea; 3 Regional Cardiocerebrovascular Disease Center, Gyeongsang National University, Jinju, South Korea; 4 Biomedical Research Institute, Gyeongsang National University, Jinju, South Korea

**Keywords:** Myopia, Risk factor, Parental myopia, Age, BMI, Near work

## Abstract

**Background:**

To evaluate the prevalence and risk factors associated with myopia and high myopia in children in South Korea.

**Methods:**

A total of 983 children 5–18 years of age who participated in the Korean National Health and Nutrition Examination Survey 2016–2017 (KNHANES VII), a nationwide population-based cross-sectional study, were evaluated. Myopia and high myopia were defined as a spherical equivalent (SE) ≤ − 0.5 diopters (D) and SE ≤ --6.0 D. The association between refractive errors and potential risk factors for myopia was analyzed.

**Results:**

The prevalence of myopia and high myopia was 65.4 and 6.9%, respectively. Older age and parental myopia were significantly associated with both myopia and high myopia, while higher body mass index (BMI) was associated with high myopia only. Although the proportion of subjects who spent more time on near work activities (≥4 h/day) was sequentially increased with increased refractive error, this tendency was not statistically significant by multivariable logistic regression.

**Conclusions:**

Korean children had a high prevalence of myopia and high myopia. In this age group, the risk of myopia increased with aging and parental myopia. Higher BMI may be associated with high myopia.

## Background

Myopia is one of the most common ocular disorders, and its prevalence has sharply increased worldwide, especially in East Asia [1]. The prevalence of myopia in children and adolescents in Korea ranges from 50% in children aged 5–11 years to 78.8% in children aged 12–18 years [2]. This prevalence is comparable to that in China (70.9% in children aged 6–18 years) [3], and higher than that in Japan (43.5% in 12-year-old children and 66.0% in 17-year-old children) [4].

The risk factors for developing myopia are multifactorial, and both genetic and environmental factors play a role in disease development and progression. Published research suggests possible risk factors including parental myopia, decreased outdoor activity, height, low serum vitamin D, higher level of education, excessive near work activity, high body mass index (BMI), and high socioeconomic status [5–11]. Uncorrected high myopia may result in amblyopia and decrease school achievement in children, while myopia itself could cause blinding ocular diseases, such as retinal detachment, myopic degeneration and myopic retinopathy [5, 12]. From this prospective, identifying the updated prevalence in Korea and risk factors of myopia and high myopia is necessary to prevent myopia and reduce the socioeconomic burden of the disease [13]. Therefore, we evaluated myopia and high myopia prevalence, and identified associations between possible risk factors for developing myopia in South Korean children and adolescents using KNHANES VII (2016–2017) data.

## Methods

### Study population

**Fig. 1:**
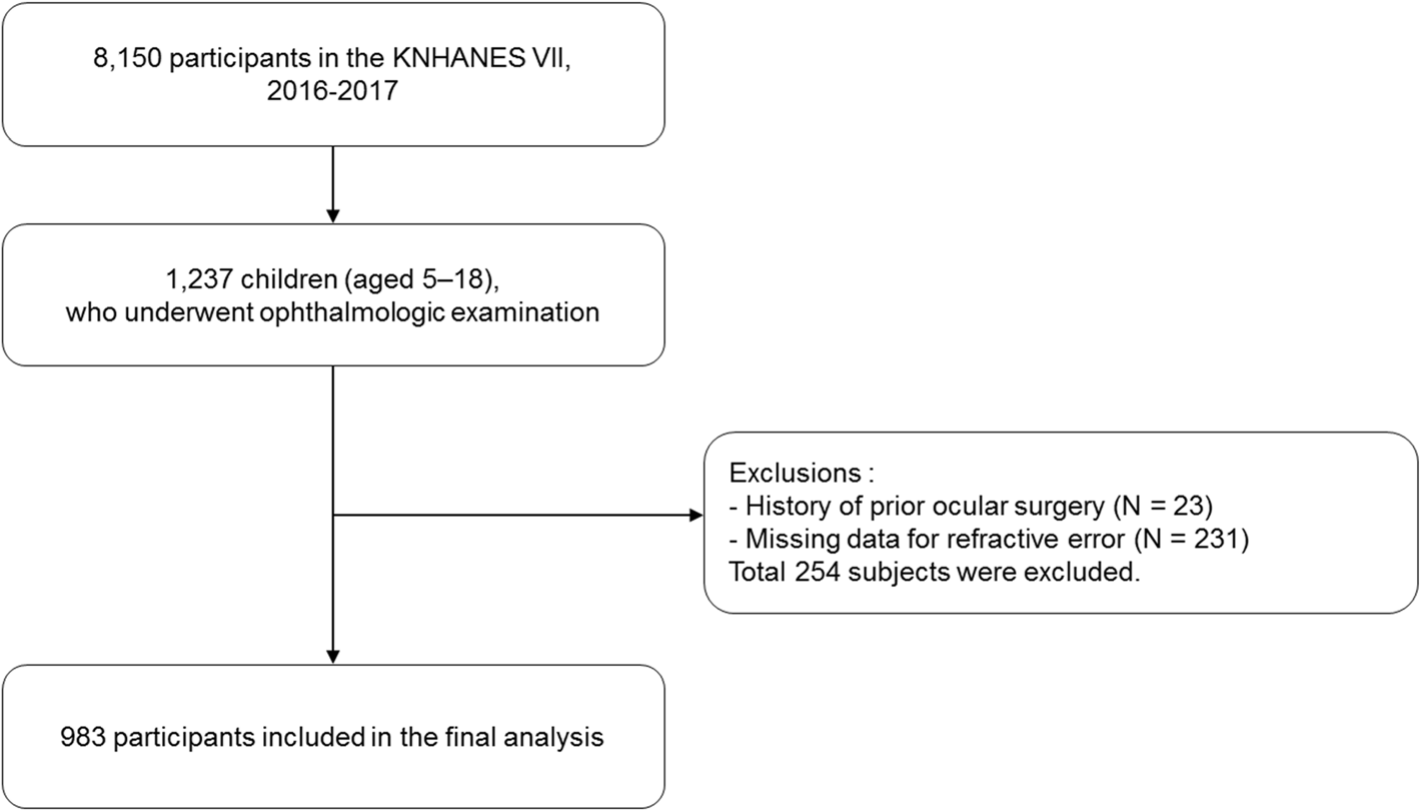
A flowchart describing the study participants included in the final analysis

The KNHANES is a nationwide, population-based, and cross-sectional health examination and survey conducted regularly since 1998 by the Division of Chronic Disease Surveillance, Korea Centers for Disease Control and Prevention, Ministry of Health and Welfare. The survey consists of a health interview survey, a nutrition survey, and a health examination survey. A stratified, multistage probability sampling design is used for the selection of household units to participate in the survey. The detailed study design of the KNHANES has been published previously [14]. In KNHANES VII, which began in 2016, out of the total source population of 8150 participants, 1237 children between 5 and 18 years of age were eligible for this study. Among eligible subjects, 23 children who had a history of prior ocular surgery or trauma and 231 children with missing data for refractive error were excluded. Finally, 983 children were analyzed, as described in Fig. 1. KNHANES VII was conducted according to the Declaration of Helsinki. This survey was reviewed by the Institutional Review Board of the Korea Centers for Disease Control and Prevention, all participants in the survey signed an informed consent form.

### Ophthalmic examinations and variable definitions

All participants underwent autorefraction in both eyes using a Topcon KR8800 autorefractor (Topcon, Tokyo, Japan) displaying a non-accommodative picture target with standard background illumination. The spherical equivalent (SE) refractive error was calculated as the sphere + 1/2 cylinder. Myopia was defined as SE ≤ -0.50 diopters (D), and high myopia was defined as SE ≤ --6.0 D [10]. Low myopia was defined as SE between − 0.50 and − 6.00.

BMI was calculated as weight (kg)/height(m)^2^; household income was categorized into quartile, with 1 (lowest) to 4 (highest). Age, gender, presence of parental myopia, time spent on near work activities, and prior history of accompanying disease (diagnosed by a doctor) were also collected.

### Statistical analysis

Statistical analyses were performed using the SAS survey procedure (version 9.2; SAS Institute, Inc., Cary, NC, USA) to reflect the complex sampling design and sampling weights of KNHANES, and to provide representative national prevalence estimates. The procedures included unequal probabilities of selection, oversampling, and non-response such that inferences could be made about Korean adult participants. KNHANES sampling was weighted by adjusting for oversampling and nonresponses [15].

The representative refractive error was defined based on the subject’s left eye [16]. Potential risk factors were assessed by subject, not by eye. Age, gender, BMI, presence of parental myopia, time spent on near work activities, household income, and accompanying disease (atopic dermatitis, allergic rhinitis, asthma, sinusitis, otitis media, Attention deficit hyperactivity disorder (ADHD)) were analyzed as possible risk factors for pediatric myopia using univariable logistic regression. Factors with *P* < 0.2 were simultaneously adjusted in a multivariable logistic regression analysis, where *P* < 0.05 was considered statistically significant.

## Results

### General subject characteristics

General subject characteristics are shown in Table 1. This study included 938 children aged 5–18 years old. The mean subject age was 12.2 ± 0.2 years, and mean refractive error was − 1.84 ± 2.38. Among the subjects, 643 (65.4%) had myopia, and 68 (6.9%) had high myopia. In a univariable analysis, the representative value (mean, median) of age and BMI sequentially increased with increased myopia level (non myopia - low myopia - high myopia), and was statistically significant (*P* < 0.001). The participants with parental myopia (*P* = 0.016) and increased time spent on near work activities (*P* = 0.033) had increased risk of myopia in a similar manner. Prior histories of accompanying disease, such as atopic dermatitis or sinusitis, as diagnosed by a doctor, were also found to be possible factors influencing the development of pediatric myopia.

**Table 1:** Characteristics of the study participants aged 5–18 years old according to refractive errors

	Non-myopia (−0.50 < SE)	Low myopia (−6.0 < SE ≤ -0.5)	High myopia (SE ≤ -6.0)	Total	*P*-value
Number	340	575	68	983	
Refractive errors (D)					
Mean (SD)	0.32 (0.85)	−2.48 (1.60)	−7.19 (0.97)	−1.84 (2.38)	
Median (IQR)	0.13 (−0.13, 0.5)	−2.13 (−3.88, − 1.00)	−7.06 (− 7.63, −6.44)	−1.13 (− 3.38, − 0.13)	
Age (years)					<.001*
Mean (SD)	9.39 (0.31)	13.02 (0.18)	15.40 (0.30)	12.16 (0.19)	
Median (IQR)	7.71 (5.65, 11.30)	13.11 (10.00, 15.40)	15.36 (13.31, 16.65)	12.18 (8.20, 15.17)	
Gender, n (%)					0.752^a^
Male	171 (50.29)	301 (52.35)	33 (48.53)	505 (51.37)	
Female	169 (49.71)	274 (47.65)	35 (51.47)	478 (48.63)	
BMI (kg/m^2^)					<.001*
Mean (SD)	17.76 (0.28)	20.01 (0.22)	22.58 (0.61)	19.57 (0.81)	
Median (IQR)	16.83 (15.17, 19.53)	19.40 (17.20, 22.41)	21.80 (19.56, 24.72)	18.90 (16.56, 21.97)	
Parental myopia, n (%)					0.016^a^
Yes	210 (61.77)	421 (73.22)	56 (82.35)	687 (69.89)	
Father	75 (35.71)	120 (28.50)	20 (35.71)	215 (31.30)	
Mother	75 (35.71)	143 (33.97)	16 (28.57)	234 (34.06)	
Both	60 (28.57)	158 (37.53)	20 (35.71)	238 (34.64)	
No	130 (38.2)	154 (26.78)	12 (17.65)	296 (30.11)	
Near work, n (%)					0.033^a^
≤ 1 h/D	60 (17.65)	50 (8.70)	4 (5.88)	114 (11.60)	
1–2 h/D	139 (40.88)	164 (28.52)	17 (25.00)	320 (32.55)	
3 h/D	68 (20.00)	133 (23.13)	12 (17.65)	213 (21.67)	
4 h/D ≤	73 (21.47)	228 (39.65)	35 (51.47)	336 (34.18)	
Household income					0.247^a^
1 (Lowest)	28 (8.24)	50 (8.70)	8 (11.76)	86 (8.75)	
2	97 (28.53)	129 (22.43)	12 (17.65)	238 (24.21)	
3	99 (29.12)	197 (34.26)	20 (29.41)	316 (32.15)	
4 (Highest)	116 (34.12)	199 (34.61)	28 (41.18)	343 (34.89)	
Accompanying disease, n (%)					
Atopic dermatitis	56 (16.47)	94 (16.35)	6 (8.82)	156 (15.87)	0.056^a^
Allergic rhinitis	86 (25.29)	168 (29.22)	24 (35.29)	278 (28.28)	0.250^a^
Asthma	10 (2.94)	29 (5.04)	3 (4.41)	42 (4.27)	0.670^a^
Sinusitis	20 (5.88)	34 (5.91)	7 (10.29)	61 (6.21)	0.154^a^
Otitis media	86 (25.29)	149 (25.91)	12 (17.65)	247 (25.13)	0.492^a^
ADHD	4 (1.18)	3 (0.52)	0 (0.00)	7 (0.71)	-^a^

All data are expressed as n (%) for categorical variables or mean (SD), median (IQR) for continuous variables

*SE* spherical equivalent; *D* diopters; *SD* standard deviation; *IQR* interquartile range; *BMI* body max index; *ADHD* attention deficit-hyperactivity disorder

^a^Rao-Scott Chi-squre

*P-values are calculated using the ANOVA

### Factors associated with myopia and high myopia

In the adjusted multivariable model, older age and parental myopia were significantly associated with myopia. According to this analysis, 1 additional year of age was associated with a 1.27-fold higher risk for myopia (95% CI, 1.18–1.37; *P* < 0.001), and a 1.44-fold higher risk for high myopia (95% CI, 1.26–1.64; *P* < 0.001), compared with children 1 year younger. In a similar manner, children with myopic parents had 1.84-fold greater risk for myopia (95% CI, 1.27–2.68; *P* = 0.002) and 3.48-fold greater risk for high myopia (95% CI, 1.28–9.46; *P* = 0.015) than children without myopic parents. Higher BMI was significantly associated with high myopia (OR, 1.19; 95% CI, 1.04–1.36; *P* = 0.009), but was not significantly associated with myopia. Logistic regression analysis identified that Increased time spent on near work activities (> 4 h/day), sinusitis, and atopic dermatitis were non-significantly associated with increased risk of myopia (Table 2).

**Table 2:** Association with risk factors for the pediatric myopia and high myopia

	Non-myopia (−0.50 < SE)	Myopia (SE ≤ -0.5)	OR (95% CI)	*P*-value	High myopia (SE ≤ -6.0)	OR (95% CI)	*P*-value
Number	340	643			68		
Age (years)			1.27 (1.18, 1.37)	<.001		1.44 (1.26, 1.64)	<.001
Mean (SD)	9.39 (0.31)	13.31 (0.16)			15.40 (0.30)		
Median (IQR)	7.71 (5.65, 11.30)	13.45 (10.39, 15.66)			15.36 (13.31, 16.65)		
BMI (kg/m^2^)			1.04 (0.97, 1.12)	0.239		1.19 (1.04, 1.36)	0.009
Mean (SD)	17.76 (0.28)	20.33 (0.21)			22.58 (0.61)		
Median (IQR)	16.83 (15.17, 19.53)	19.77 (17.37, 22.63)			21.80 (19.56, 24.72)		
Parental myopia, n (%)	210 (61.76)	477 (74.18)	1.84 (1.27, 2.68)	0.002	56 (82.35)	3.48 (1.28, 9.46)	0.015
Near work, n (%)							
≤ 1 h/D	60 (51.25)	54 (48.75)	Reference		4 (5.88)	Reference	
1–2 h/D	139 (38.56)	181 (61.44)	1.05 (0.6, 1.85)	0.856	17 (25.00)	0.98 (0.31, 3.09)	0.970
3 h/D	68 (24.64)	145 (75.36)	1.53 (0.86, 2.7)	0.144	12 (17.65)	0.77 (0.18, 3.22)	0.717
4 h/D ≤	73 (18.85)	263 (81.15)	1.38 (0.77, 2.49)	0.282	35 (51.47)	1.12 (0.35, 3.56)	0.852
Accompanying disease, n (%)							
Atopic dermatitis	56 (16.47)	100 (15.55)	1.04 (0.72, 1.5)	0.838	156 (15.87)	0.62 (0.21, 1.9)	0.403
Sinusitis	20 (5.88)	41 (6.38)	0.70 (0.28, 1.75)	0.441	61 (6.21)	0.63 (0.11, 3.44)	0.588

All data are expressed as n (%) for categorical variables or mean (SD), median (IQR) for continuous variables

*SE* spherical equivalent; *OR* odds ratio; *CI* confidence interval; *SD* standard deviation; *IQR* interquartile range; *BMI* body max index;

*P*-values are calculated using the multivariavle logistic regression

### Overall prevalence of myopia by age

**Fig. 2:**
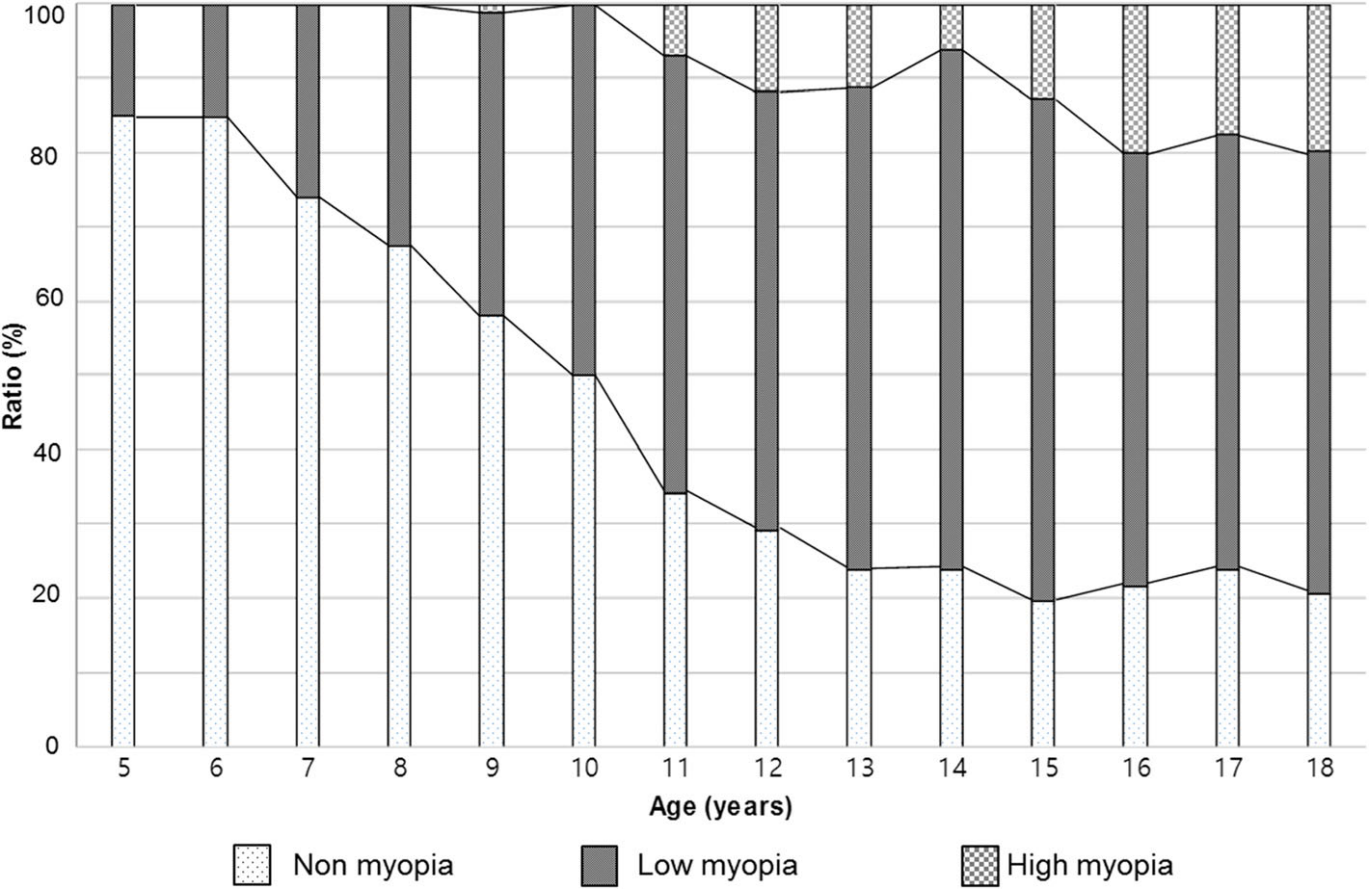
Overall prevalence of myopia by age (5–18 year old), grouped non myopia, low myopia, high myopia

The proportion of myopic children was 15.0% at 5 year, 15.2% at 6 year old age group. It was sharply increased from 7 year old age, reached 76% at 13 year old age group, showed little change after this age. That of high myopic children began to increase from 6.8% at 11 year old age, stabilized after 16 year old age, reached about 20% (Fig. 2).

## Discussion

The present study identified a high prevalence of myopia (65.4%) and high myopia (6.9%) in Korean children between 5 and 18 years of age. Older age and parental myopia were strongly associated with myopia and high myopia, while higher BMI was significantly associated with high myopia only. Other possible risk factors, such as time spent on near work activities, household income, and associated diseases, were not significantly associated with prevalence of myopia or high myopia.

An ophthalmologic examination was first included in KNHANES IV, 2008. Several studies using KNHANES IV–V (2008–2012) data presented a myopia growth chart [17], and identified a strong relationship between parental myopia and child myopia [18]. In the present study, we used pediatric data in KNHANES VII, the most up-to-date cross-sectional nationwide ophthalmologic data set. Further, this data set includes information regarding parental myopia and near work time, which was not included in prior investigations. In consideration of the inherent limitations of a cross-sectional study and compounding bias among multiple factors affecting development of myopia, the results of each study should be interpreted relative to prior and subsequent KNHANES data, and data from other Asian countries in similar periods.

The prevalence of pediatric myopia and high myopia in the present study is comparable to that in other studies using KNHANES IV–V (64.6–66.2%, 5.4–5.9%, respectively) [10, 17, 18] and that in China (70.9, 8.6%, respectively) [3], and is higher than that in Taiwan (36.4%) [19]. The prevalence of myopia in young Korean adults sharply increased to 78.9% in adults aged 20–29, 60.7% in adults aged 40–49, and 16.1% in adults aged 60–69 in KNHANES IV–V (2008–2012) [20]. Similar results were reported in a recent study using KNHANES VI (2013–2014), with the prevalence being 81.2% in adults aged 20–29 and 60.3% in adults aged 40–49 [13]. However, the prevalence of adult high myopia has increased from 10.9 to 11.2% in adults aged 20–29, and 4.1 to 5.7% in adults aged 40–49. This increase of the high myopic young adult population is predictable considering the change of high myopia in children, where the high myopic proportion increased from 12.2% (2008–2012) to 19.6% (2016–2017) in 18-year-old adolescents (Fig. 2). High myopia may cause critical vision-threatening pathologies such as retinal detachment, glaucoma, and maculopathy [12, 21, 22]. An effort to reduce the high myopic population in children and adolescents is necessary, including identification of modifiable risk factors for myopia and high myopia.

Age is one of the most important risk factors related to myopia. As identified in the previous literature, [17] axial eyeball growth in Korean children is most accelerated in children 7.5–11.9 years of age, and ends at 16 years of age. In the present study, the proportion of myopia was stabilized after 13 years of age, while that of high myopia continued to increase until 16 years of age (Fig. 2). Generally, in early onset myopia that progresses to high myopia, eyeball elongation in high myopic children continues to progress 2–3 years later than that in myopic children without high myopia, which would be consistent with the continued increase in high myopic children.

Parental myopia was identified as the greatest contributing risk factor in the present study. Children with myopic parents had a 1.84-fold increased risk of myopia compared with children with non-myopic parents. In high myopia, this tendency increased to a 3.48-fold increased risk of high myopia compared with children with non-myopic parents. The strong association between parental and child myopia is well established, [8, 18, 23] with an even greater risk in children with two myopic parents than in children with one or no myopic parent. In this study, the mean refractive error was − 2.63 ± 2.72 in children with one or more myopic parents, and − 2.97 ± 2.01 in children with two myopic parents, relative to the entire mean value, − 1.84 ± 2.38. As mentioned above, young adults in Korea tend to have more myopia than older adults, so their children would be expected to have a greater myopic refractive error. Therefore, the prevalence of myopia and high myopia could rapidly increase in the next generation.

The present study identified a significant relationship between higher BMI and high myopia. The effect of BMI on development of myopia is still controversial. Saw et al. reported that children who were heavier or who had a higher BMI tended to have more hyperopic eyes with shorter vitreous chambers [7]. Some studies of the adult population also reported that persons with a higher BMI tend to have more hyperopic eyes [11, 24, 25]. These studies found that BMI was not associated with ocular parameters, and did not explain the causative association of hyperopia with lower BMI. Meanwhile, recent studies reported that higher BMI is related to developing myopia in children [3, 18]. Obese children who engage in fewer outdoor activities or have increased near work activity are more at risk for myopic refractive error [18]. However, when near work activity time was included in multivariable models in the present study, the effect of higher BMI on high myopia was still significant (OR, 3.48; *P* = 0.009). Another possible explanation is that high BMI is a marker of higher socioeconomic status, which is itself a risk factor for myopia, although the correlation was statistically insignificant in the present study. BMI is independent of height, and is a better index of body fat than weight. Recent studies have identified that child growth rate is accelerated with early puberty in girls, [26] and Gardiner [27] found that body weight growth rate of school-age children was faster in children with myopia. This could explain the discrepancy with studies specifically examining myopia in males, [11, 28], which reported that higher BMI was not related to myopia.

The time spent on near work activities is a newly adopted factor in the KNHANES VII survey. Although the proportion of subjects who spent more time on near work activities (≥4 h/day) was sequentially increased with increased refractive error, the effect was weakened after adjusting for other risk factors. However, this tendency remained with subgroup analysis (≥3 h/day, ≥2 h day) This factor is associated with both myopia and high myopia in the adult population [13], but was not associated with pediatric myopia in the present study. Saw et al. [6] reported that children who read > 2 books per week had longer axial lengths (+ 0.17 mm) than children who read ≤2 books a week, whereas Mutti et al. [5] reported that near work was not associated with myopia risk.

Time spent on near work activities, outdoor activities, and sun exposure are inter-related. Serum vitamin D is often considered a biomarker of outdoor activity [29]. However, the relationship between near work-induced transient myopia (NITM) and permanent myopia has remained somewhat indirect and elusive, [9] as the additivity of NITM was not found following sequences of interrupted near tasks [30]. Providing rest periods between each near task trial appears to prevent a cumulative effect, and therefore decreases the probability of developing myopia. The weak correlation of near work activity time with myopia in the present study suggest that the effect of near work is a compounding factor inversely related to outdoor activity, rather than an independent risk factor.

There are several limitations in the present study. Refractive errors were not evaluated under cycloplegic conditions, which could bias the results in younger subjects, who tended to have a more active accommodative response than older subjects. However, the association of the refractive errors and aging, parental myopia are robust and consistent throughout all analyzes, the difference between auto-refraction and cycloplegic refraction is closely related within 1 diopter according to the previous report [31]. This level of agreement is not surprising because, while lack of cycloplegia leads to an over-estimation of myopia, the biggest problem with noncycloplegic refractions concern the under-estimation of hyperopia and the resulting errors in estimation of mean spherical equivalent. Even then, the cycloplegic refraction is definitely required to assess refractive error in the children, the researchers should continue their efforts to get the exact results with cycloplegic refractions in this type of studies. Second, the KNHANES is a cross-sectional study, so the results cannot guarantee a causal relationship. However, findings identified in this study are consistent with KNHANES IV–VI. Therefore, data interpretation relative to recent KNHANES data could provide a new trend analysis, such as an increase in high myopic children. Third, possible risk factors such as parental education level, serum vitamin D level, WBC counts, outdoor activity, daily sun exposure, and anthropometric data were not evaluated. Lastly, ethnic and regional differences should be considered to generalize these results to other populations.

## Conclusion

We identified a high prevalence of myopia and high myopia in a large, well-controlled sample of Korean children. In this age group, the risk of myopia and high myopia increased with aging and parental myopia, and BMI may have been associated with high myopia. Further studies are required to reveal causal relationships, and adjusted risk evaluation with the inter-related factors of near work activity time, sun exposure time, outdoor activity time, and serum vitamin D level is necessary.

## Data Availability

The all raw data of survey is available http://knhanes.cdc.go.kr/. The datasets during and/or analyzed during the current study available from the corresponding author on reasonable request.

## Abbreviations

ADHD: Attention deficit-hyperactivity disorder
BMI: Body mass index
D: Diopters
KNHANES: Korean National Health and Nutrition Examination Survey
SE: Spherical equivalent
